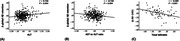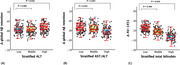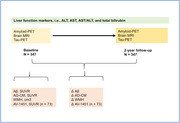# Influence of liver function on Alzheimer’s brain pathologies: A longitudinal study

**DOI:** 10.1002/alz.088641

**Published:** 2025-01-03

**Authors:** Jee Wook Kim, Min Soo Byun, Dahyun Yi, Joon Hyung Jung, Nayeong Kong, Yoon Young Chang, Gijung Jung, Hyejin Ahn, Jun‐Young Lee, Koung Mi Kang, Chul‐Ho Sohn, Yun‐Sang Lee, Yu Kyeong Kim, Dong Young Lee, KBASE Research Group

**Affiliations:** ^1^ Hallym University Dongtan Sacred Hospital, Hwaseong, Gyeonggi‐do Korea, Republic of (South); ^2^ Department of Psychiatry, Seoul National University College of Medicine, Seoul Korea, Republic of (South); ^3^ Seoul National University Hospital, Seoul Korea, Republic of (South); ^4^ Department of Neuropsychiatry, Seoul National University Hospital, Seoul Korea, Republic of (South); ^5^ Institute of Human Behavioral Medicine, Medical Research Center, Seoul National University, Seoul Korea, Republic of (South); ^6^ Department of Psychiatry, Chungbuk National University Hospital, Cheongju Korea, Republic of (South); ^7^ Department of Psychiatry, Keimyung University Hospital, Daegu Korea, Republic of (South); ^8^ Inje University Sanggye Paik Hospital, Seoul Korea, Republic of (South); ^9^ Interdisciplinary program of cognitive science, Seoul National University, Seoul Korea, Republic of (South); ^10^ SMG‐SNU Boramae Medical Center, Seoul Korea, Republic of (South); ^11^ Department of Neuropsychiatry, SMG‐SNU Boramae Medical Center, Seoul Korea, Republic of (South); ^12^ Department of Radiology, Seoul National University Hospital, Seoul Korea, Republic of (South); ^13^ Seoul National University College of Medicine, Seoul Korea, Republic of (South); ^14^ Department of Nuclear Medicine, Seoul National University College of Medicine, Seoul Korea, Republic of (South); ^15^ Department of Nuclear Medicine, SMG‐SNU Boramae Medical Center, Seoul Korea, Republic of (South); ^16^ Seoul National University, Seoul Korea, Republic of (South); ^17^ Seoul National University Dementia Research Center, Seoul Korea, Republic of (South)

## Abstract

**Background:**

Numerous studies have demonstrated an association between altered liver function and Alzheimer’s disease (AD) or related dementia. Nevertheless, the neuropathological substrates underlying the association remains unclear. Our aim was to investigate the association between liver function markers and the longitudinal changes of *in vivo* AD pathologies.

**Methods:**

We utilized a longitudinal study designs using data from the Korean Brain Aging Study for Early Diagnosis and Prediction of Alzheimer’s Disease, an ongoing prospective cohort study that began in 2014. Total 347 older adults between 55 and 90 years of age assessed at both baseline and 2‐year follow‐up were included in community and memory clinic setting. We used four liver function markers (alanine aminotransferase [ALT], aspartate aminotransferase [AST], AST to ALT ratio, and total bilirubin) as exposure variables. Participants underwent comprehensive clinical assessments and multimodal brain imaging, including Pittsburgh compound‐B positron emission tomography (PET), AV‐1451 PET, fluorodeoxyglucose (FDG)‐PET, and MRI at both baseline and the 2‐year follow‐up.

**Results:**

A higher ALT level and lower AST to ALT ratio at baseline were associated with greater amyloid deposition over a 2‐year period (ALT: *β*, 0.151 [95% CI, 0.007 to 0.033], *P* = 0.004; AST to ALT ratio: *β*, ‐0.153 [95% CI, ‐0.050 to ‐0.010], *P* = 0.004), but not with the change of other neuroimaging markers. A lower total bilirubin level was associated with greater tau deposition over a 2‐year period (*β*, ‐0.442 [95% CI, ‐0.385 to ‐0.106], *P* < 0.001), but not with the change of other neuroimaging markers. In contrast, AST alone was not associated with any neuroimaging marker change.

**Conclusions:**

The present findings, based on longitudinal observation for older adults, suggest that lower liver function may accelerate the accumulation of AD pathology in the brain. These results also support the possibility that the liver‐brain axis could be a potential target for therapeutic or preventive strategies against AD.